# Detection of serum FOXM1 and IGF2 in patients with ARDS and their correlation with disease and prognosis

**DOI:** 10.1515/med-2024-1093

**Published:** 2024-12-18

**Authors:** Chao Liu, Shengrui Zhang, Weiwei Zhang, Jinfeng Wang

**Affiliations:** Department of Intensive Care Unit, Ganzhou People’s Hospital, Ganzhou, Jiangxi, 341000, China; Department of Oncology, The First Affiliated Hospital of Gannan Medical University, No. 23, Qingnian Road, Zhanggong District, Ganzhou, Jiangxi, 341000, China

**Keywords:** acute respiratory distress syndrome, forkhead box protein M1, insulin-like growth factor 2, oxygenation index, prognosis

## Abstract

**Objective:**

To investigate the relationship between the expression levels of serum forkhead box protein M1(FOXM1) and insulin-like growth factor 2 (IGF2) mRNA in patients with acute respiratory distress syndrome (ARDS) condition and prognosis.

**Methods:**

Ninety patients with ARDS admitted to our hospital were regarded as the ARDS group, according to the prognosis, they were grouped into death group (*n* = 64) and survival group (*n* = 126); the control group consisted of 190 healthy individuals.

**Results:**

Compared with the control group, the level of serum FOXM1 mRNA in ARDS group was obviously lower, and the level of IGF2 mRNA was higher. The serum IGF2 mRNA, serum creatinine, inhaled oxygen concentration (FiO_2_), and mechanical ventilation time in the death group were higher than those in the control group, and the arterial oxygen partial pressure (PaO_2_), FOXM1 mRNA, and oxygenation index (PaO_2_/FiO_2_) were lower than those in control group. Logistic regression analysis indicated that FOXM1, IGF2, and PaO_2_/FiO_2_ were significant factors influencing the prognosis and mortality in ARDS patients. Correlation analysis showed that there was a negative correlation between serum FOXM1 and IGF2 mRNA levels in patients with ARDS.

**Conclusion:**

Serum FOXM1 and IGF2 mRNA in patients with ARDS are correlated with the severity and prognosis of ARDS.

## 1 Introduction

In acute respiratory distress syndrome (ARDS), multiple pathogenic factors including trauma, pneumonia, sepsis incur lung injury, leading to acute respiratory failure and persistent hypoxemia [1]. With complicated etiology, ARDS has a high fatality rate despite the improvement of clinical respiratory support technology and the continuous update in diagnosis and pulmonary re-expansion technique [2]. Hence, efficient and unique prognostic indexes will help start early symptomatic treatment and promote the outcome of the disease. Previous studies verify that the pathological mechanism of ARDS closely correlates with inflammatory response. ARDS is mainly caused by various inflammatory mediators that activate the inflammatory cascade and induce diffuse lung parenchymal injury [3]. Fork head box M1 (FOXM1), a member of the FOX transcription factor family, acts as an important regulator of cell cycle. By direct or indirect activation of target gene transcription expression, it takes part in multiple pathophysiological processes such as DNA damage repair and angiogenesis, which is indispensable in normal lung development [4]. Recent scholars found that the overexpression of FOXM1 enhances the therapeutic effects of bone marrow mesenchymal stem cells in treating ARDS [5]. Insulin-like growth factor 2 (IGF2) is a multifunctional growth hormone that regulates cell activity. Abnormally high expression of IGF2 has been proved to facilitate lipopolysaccharide (LPS)-induced inflammatory damage and aggravate acute pneumonia [6]. It is noteworthy that IGF2 knockout can inhibit the secretion of inflammatory cytokines to reduce LPS-induced cell damage in acute pneumonia [7]. It is thus speculated that FOXM1 and IGF2 are involved in the progression of ARDS, but their correlation in ARDS remains unclear. This study aims to investigate the serum levels of FOXM1 and IGF2 mRNA in ARDS patients, analyze its relationship with the disease, and assess its prognostic prediction value.

## 2 Data and methods

### 2.1 General data

A total of 190 ARDS patients (ARDS group) admitted to Ganzhou People’s Hospital from January 2020 to December 2022 were retrospectively selected, including 105 males and 85 females aged 30–70 (55.75 ± 6.84) years, with body mass index (BMI) in the range of 18–26 (22.73 ± 2.06) kg/m^2^. Inclusion criteria were (1) meet the diagnostic criteria for ARDS [8], (2) onset time <5 days, and (3) complete clinical data. Exclusion criteria were (1) with pulmonary diseases such as pulmonary tuberculosis, cardiogenic edema, lung tumors, and chronic obstructive pulmonary disease; (2) with contraindications of mechanical ventilation; (3) administration of hormone drugs 3 months before admission; (4) with respiratory failure due to fluid overload or heart failure; and (5) related to COVID-19. This study was approved by the hospital ethics committee. The 190 healthy volunteers in physical examination of our hospital during the same period were the control group, including 96 males and 94 females aged 31–69 (54.39 ± 7.54) years, with BMI in the range of 18–26 (22.08 ± 2.27) kg/m^2^. The above data have no significant difference between the control group and ARDS group (*P* > 0.05).

**Figure 1:**
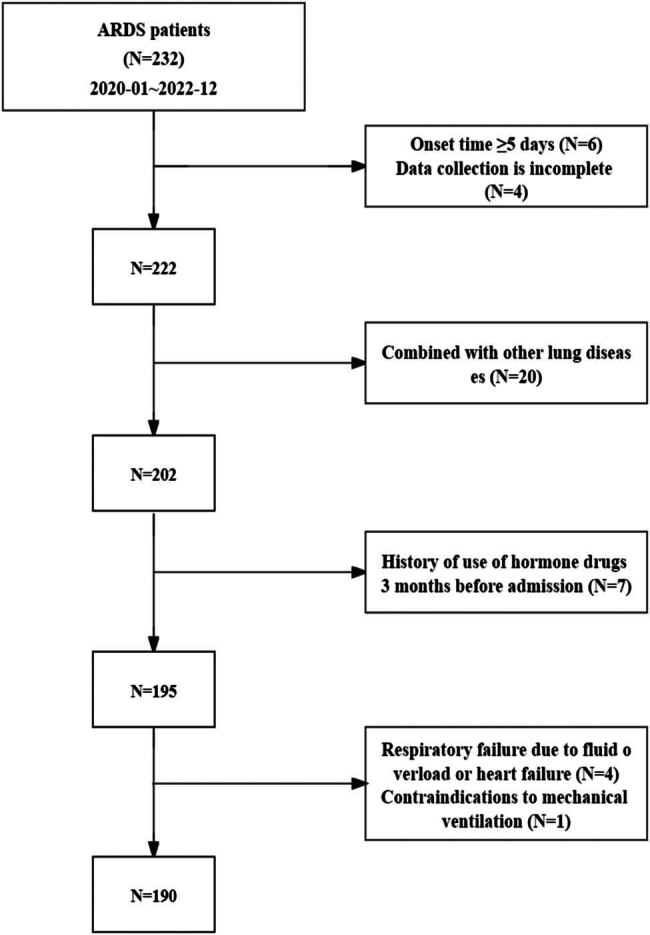
Study flow chart.

According to clinical outcome within 28 days of hospitalization (defined as ICU or in-hospital death), the ARDS patients were divided into death group (*n* = 64) and survival group (*n* = 126). According to the oxygenation index (PaO_2_/FiO_2_) within 24 h after admission, the 190 ARDS were divided into three groups in light of the disease severity, with >200 mmHg for mild group (*n* = 59), 100–200 mmHg for moderate group (*n* = 76), and ≤100 mmHg for severe group (*n* = 55) (Figure 1).

### 2.2 Methods

#### 2.2.1 Collection of clinical baseline data

Basic data of ARDS patients were collected, including general demographic data (age, etc.), heart rate, diabetes history, respiratory rate, hypertension history, biochemical indexes, ARDS etiology (pulmonary embolism, sepsis, trauma, etc.), blood gas indexes, mechanical ventilation duration, etc. The biochemical indexes of serum creatinine (Scr), albumin (Alb), and urate nitrogen (BUN) were detected by automatic biochemical analyzer (Beckmann AU680). Arterial blood gas analyzer (Danish ray ABL80) was used for blood gas analysis. The indexes included arterial partial pressure of carbon dioxide (PaCO_2_), arterial partial pressure of oxygen (PaO_2_), inhaled oxygen concentration (FiO_2_), and calculated PaO_2_/FiO_2_.

#### 2.2.2 Detection of serum FOXM1 and IGF2 levels

Fasting peripheral blood was collected from ARDS group at admission and control group during physical examination. The serum was collected by centrifugation, and the serum expressions of FOXM1 and IGF2 mRNA were detected by real-time quantitative PCR (qRT-PCR). RNA extraction kit (article No: 9112) was used to extract total RNA from serum samples, and RNA concentration and purity were determined by spectrophotometer (NanoDrop ND-1000, Kailebo (Beijing) Technology Development Co., LTD). After obtaining qualified RNA samples, cDNA was extracted using a reverse transcription kit (article No. 639504) and qRT-PCR expression analysis was performed according to the instructions of the TB Green^®^Premix Ex Taq™II kit (article No. RR820A), with sequence shown in Table 1. β-actin was used as the internal parameter of FOXM1 and IGF2. The relative expression levels of FOXM1 and IGF2 mRNA were analyzed by 2^−ΔΔCt^. All reagents and primers were provided by TaKaRa Biomedical Technology (Beijing) Co., Ltd.

**Table 1:** Primer sequence

Gene	Sequence
FOXM1	Forward: 5′-ATACGTGGATTGAGGACCACT-3′
	Reverse: 5′-TCCAATGTCAAGTAGCGGTTG-3′
IGF2	Forward: 5′-CTTGGACTTTGAGTCAAATTGG-3′
	Reverse: 5′-GGTCGTGCCAATTACATTTCA-3ʹ
β-actin	Forward: 5ʹ-TGGAATCCTGTGGCATCCATGAAAC-3″
	Reverse: 5ʹ-ACGCAGCTCAGTAACAGTCCG-3″

### 2.3 Statistical analysis

SPSS 25.0 software was used. Statistical data including gender, etiology, and smoking were represented as *n* (%) and tested by *χ* ^2^. Measurement data such as FOXM1 and IGF2 mRNA were expressed as (*x̅* ± *s*). *t* test was used if two groups were involved, one-way analysis of variance was used when three groups were involved, and SNK-*q* test was used for pairwise group comparison. Pearson coefficient was used to analyze the correlation between FOXM1 and IGF2 mRNA levels. Logistic regression was used to identify prognostic factors of ARDS patients. The receiver operating characteristic (ROC) curve was taken to evaluate the efficacy of serum FOXM1 and IGF2 mRNA in predicting prognosis of ARDS patients, and the area under the curve (AUC) difference was compared by *Z*-test. *P* < 0.05 indicates statistically significant difference.


**Informed consent:** Informed consent was obtained from the patient or their guardian, with signatures on the consent form.
**Ethical approval:** This study involving human participants was in accordance with the ethical standards of the Medical Ethics Committee of the Ganzhou People's Hospital and with the 1964 Helsinki Declaration.

## 3 Results

### 3.1 Comparison of serum FOXM1 and IGF2 mRNA levels between ARDS group and control group

Compared with the control group, ARDS group had significantly decreased serum FOXM1 mRNA level and increased serum IGF2 mRNA level (*P* < 0.05), as shown in Table 2.

**Table 2:** Comparison of serum FOXM1 and IGF2 mRNA levels between ARDS group and control group (*x̅* ± *s*)

	ARDS group (*n* = 190)	Control group (*n* = 190)	*t*	*P*
FOXM1 mRNA	0.66 ± 0.20	1.12 ± 0.21	21.864	0.000
IGF2 mRNA	2.29 ± 0.62	1.04 ± 0.16	26.909	0.000

### 3.2 Comparison of serum FOXM1 and IGF2 mRNA levels in patients with different disease severity

Serum FOXM1 mRNA level was increased in turn in severe group, moderate group, and mild group, with serum IGF2 mRNA level decreased successively in the three groups, showing statistically significant difference (*P* < 0.05), as shown in Table 3.

**Table 3:** Comparison of serum FOXM1 and IGF2 mRNA levels in patients with different disease levels (*x̅* ± *s*)

	Mild group (*n* = 59)	Moderate group (*n* = 76)	Severe group (*n* = 55)	*F*	*P*
FOXM1 mRNA	0.85 ± 0.24	0.64 ± 0.21^a^	0.48 ± 0.14^ab^	47.826	0.000
IGF2 mRNA	1.30 ± 0.41	1.78 ± 0.44^a^	2.57 ± 0.68^ab^	88.705	0.000

Note: Compared with mild group, ^a^ *P* < 0.05; compared with moderate group, ^b^ *P* < 0.05.

### 3.3 Comparison of clinical data of patients with different prognosis

PaO_2_/FiO_2_ and PaO_2_ were lower in the death group than in the control group, while Scr, FiO_2_, and mechanical ventilation duration were higher in the former than in the latter (*P* < 0.05), as shown in Table 4.

**Table 4:** Comparison of clinical data of patients with different prognosis [(*x̅* ± *s*)/*n* (%)]

Clinical data	Death group (*n* = 64)	Survival group (*n* = 126)	*t/χ* ^2^	*P*
Gender (male/female)	37/27	68/58	0.254	0.614
Age (years)	56.18 ± 7.04	55.53 ± 6.73	0.620	0.536
BMI (kg/m^2^)	22.45 ± 2.27	22.87 ± 1.95	1.326	0.186
Smoking history	24 (37.50)	48 (38.10)	0.006	0.936
Drinking history	15 (23.44)	22 (17.46)	0.967	0.325
Diabetes mellitus	7 (10.94)	16 (12.70)	0.124	0.725
Hypertension	9 (14.06)	25 (19.84)	0.965	0.326
Pathogenesis				
Wound	17 (26.56)	31 (24.60)	2.480	0.479
Pneumonia	26 (40.63)	65 (51.59)		
SEPSIS	14 (21.88)	21 (16.67)		
Pulmonary embolism	7 (10.94)	9 (7.14)		
Mechanical ventilation time (d)	9.04 ± 1.88	6.75 ± 1.46	8.523	0.000
Respiratory rate (time/min)	25.39 ± 4.85	24.47 ± 4.66	1.227	0.221
Heart rate (time/min)	105.09 ± 15.49	103.62 ± 16.63	0.589	0.557
PaO_2_ (mmHg)	65.73 ± 7.02	72.65 ± 3.77	8.847	0.000
FiO_2_ (%)	49.05 ± 5.56	40.03 ± 3.10	14.359	0.000
PaCO_2_ (mmHg)	49.91 ± 6.24	48.51 ± 5.49	1.586	0.115
PaO_2_/FiO_2_ (mmHg)	134.01 ± 38.17	181.49 ± 54.28	6.253	0.000
BUN (mmol/L)	15.22 ± 3.08	14.56 ± 2.94	1.439	0.152
Scr (μmol/L)	186.39 ± 14.92	161.04 ± 11.73	12.815	0.000
Alb (g/L)	36.84 ± 3.47	35.92 ± 3.01	1.890	0.060

### 3.4 Comparison of serum FOXM1 and IGF2 mRNA levels in patients with different prognosis

Compared with the survival group, the death group had decreased serum FOXM1 mRNA level and increased serum IGF2 mRNA level (*P* < 0.05), as shown in Table 5.

**Table 5:** Comparison of serum FOXM1 and IGF2 mRNA levels in patients with different prognosis (*x̅* ± *s*)

	Death group (*n* = 64)	Survival group (*n* = 126)	*t*	*P*
FOXM1 mRNA	0.50 ± 0.16	0.74 ± 0.22	7.745	0.000
IGF2 mRNA	2.83 ± 0.71	2.02 ± 0.57	8.505	0.000

### 3.5 Correlation between serum FOXM1 and IGF2 mRNA levels in ARDS patients

**Figure 2:**
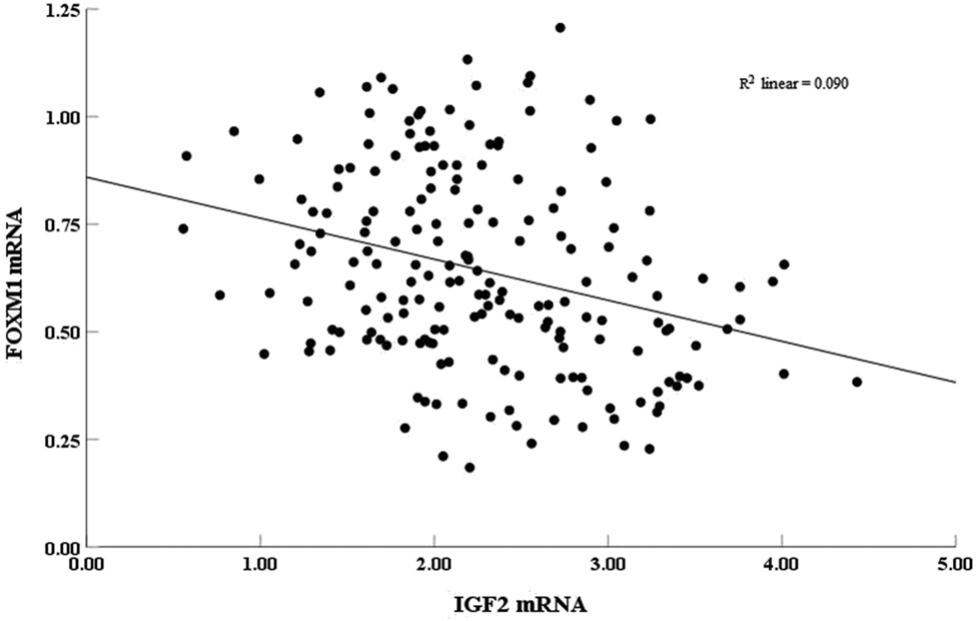
Correlation of serum FOXM1 and IGF2 mRNA levels in ARDS patients.

The correlation analysis showed that serum FOXM1 was negatively correlated with IGF2 mRNA levels in ARDS patients (*r* = −0.358, *P* = 0.000), as shown in Figure 2.

### 3.6 Logistic regression analysis of multiple factors influencing prognosis in ARDS patients

The prognosis of ARDS patients was taken as the dependent variable (death = 1, survival = 0), and the significant difference factors in univariate analysis were used as independent variables, including mechanical ventilation duration (measured value), FiO_2_ (measured value), PaO_2_ (measured value), PaO_2_/FiO_2_ (measured value), Scr (measured value), serum FOXM1 (measured value), and IGF2 (measured value). The multivariate logistic regression model was established. The results showed that FOXM1, IGF2, and PaO_2_/FiO_2_ were the factors influencing prognostic death of ARDS patients (*P* < 0.05), as shown in Table 6.

**Table 6:** Logistic regression analysis of multiple factors influencing prognosis of ARDS patients

Factors	B	SE	Wald	*P*	OR	95% CI
Mechanical ventilation time	0.160	0.159	1.007	0.316	1.173	0.859–1.602
FiO_2_	0.230	0.166	1.921	0.165	1.259	0.909–1.743
PaO_2_	−0.028	0.095	0.089	0.765	0.972	0.807–1.170
PaO_2_/FiO_2_	−0.180	0.071	6.450	0.011	0.835	0.726–0.956
Scr	0.268	0.184	2.117	0.146	1.307	0.911–1.875
FOXM1 mRNA	−0.179	0.075	5.704	0.017	0.836	0.722–0.968
IGF2 mRNA	0.583	0.158	13.631	0.000	1.792	1.314–2.442

### 3.7 Predictive efficacy of serum FOXM1 and IGF2 mRNA on ARDS patients’ prognosis

**Figure 3:**
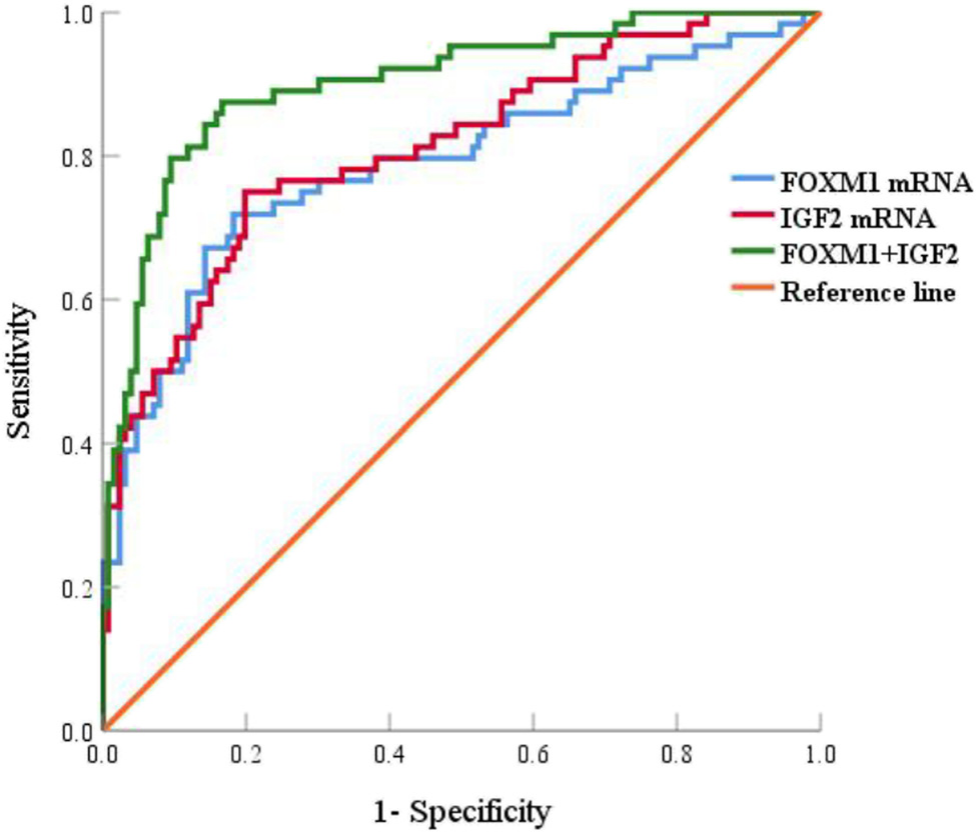
ROC curve of serum FOXM1 and IGF2 mRNA predicting prognosis of ARDS patients.

The cut-off values in prognostic death prediction of ARDS patients by serum FOXM1 and IGF2 mRNA were 0.61 and 2.39, respectively, and the AUC in prognostic death prediction by serum FOXM1 mRNA combined with IGF2 mRNA was 0.902, which was superior to 0.789 and 0.812 (*Z*/*P* = 3.161/0.002, 3.278/0.001) in single detection, as shown in Table 7 and Figure 3.

**Table 7:** Predictive efficacy of serum FOXM1 and IGF2 mRNA on prognosis of ARDS patients

	AUC	95% CI	Critical value	Sensitivity (%)	Specificity (%)	Youden index
FOXM1 mRNA	0.789	0.715–0.864	0.61	71.90	81.70	0.536
IGF2 mRNA	0.812	0.745–0.878	2.39	75.00	80.20	0.552
FOXM1+IGF2	0.902	0.854–0.950	—	87.50	81.00	0.685

## 4 Discussion

### 4.1 Background introduction of ARDS

ARDS as a common severe respiratory system disease in clinical practice has complex pathogenic factors and high morbidity and mortality [9,10]. This study retrospectively analyzed 190 ARDS patients, finding that the prognostic death rate was as high as 33.68%, which suggested a high death rate of ARDS in our center, so attention was needed. Previous scholars found that lung injury severity score, APACHE II score, etc., had certain value in judging prognosis, but the results were subjective [11]. Therefore, it is necessary to find prognostic indexes for ARDS patients to provide early intervention and increase the survival rate.

### 4.2 Correlation between FOXM1 and ARDS

Studies have shown that the pathogenesis of ARDS is inseparable from inflammatory response. Inflammatory factors secreted in large amounts break the original anti-inflammatory and pro-inflammatory system of the body, activate inflammatory mediators, damage alveolar epithelium, increase capillary permeability, aggravate lung injury, and accelerate ARDS progression [9].

Located in human chromosome 12p13.3, FOXM1 is a key factor in regulating cell cycle. Several previous studies have verified that abnormal expression of FOXM1 correlates with lung diseases such as acute lung injury, asthma, pulmonary arterial hypertension [4]. Wang et al. [12] found that FOXM1 had abnormal expression in the serum of pneumonia patients, and miR-370-3p alleviated LPS-induced lung injury by targeting FOXM1. In this study, FOXM1 mRNA had decreased expression level in the serum of ARDS patients, suggesting the important role of FOXM1 in the pathogenesis of ARDS. Previous studies have shown that, by activating Wnt/β-catenin signaling pathway, transplantation of BMSC with FOXM1 overexpression can inhibit inflammation (by reducing white blood cell count, total protein concentration, inflammatory cytokines, etc.) and apoptosis, partially restore vascular integrity, thus preventing LPS-induced ARDS [13]. Relevant studies also confirmed that endothelial cell FOXM1 was a key endogenous mediator in BMPC-induced anti-inflammatory lung injury, which can restore vascular integrity, speed up the regression of inflammation, thus contributing to the recovery of inflammatory lung injury [14]. This study showed that FOXM1 mRNA level decreased with the disease aggravation, and the FOXM1 mRNA level was lower in the death group than in the survival group, suggesting that FOXM1 was closely related to the progression and prognosis of ARDS. It was speculated that decreased FOXM1 expression would lead to reduced inhibition of inflammatory response, massive secretion of inflammatory factors and mediators, which then promote acute lung inflammation and exacerbate lung damage. It is worth noting that the abnormal expression of FOXM1 in several malignant tumors is associated with poor prognosis, and targeting FOXM1 is considered as a potential therapeutic target for improving prognosis [15]. The regression analysis in this study showed that FOXM1 was a factor influencing prognosis of ARDS, confirming that FOXM1 participates in the progression of ARDS, so monitoring its level changes can provide clinical early warning.

### 4.3 Correlation between IGF2 and ARDS

IGF2, an imprinted gene located on chromosome 7, is an acidic peptide formed by 67 amino acids expressed in tissues like liver, which can regulate cell proliferation and differentiation. Previous reports on IGF2 mostly focused on tumors, and it was found to promote disease progression as an oncogene [16]. Recently, scholars have found that IGF2 also participated in lung-related diseases. Zhang et al. [6] found that acute pneumonia patients had increased serum expression level of IGF2, while miR-370-3p inhibited LPS-induced apoptosis and inflammatory damage by targeting IGF2. LPS-treated WI-38 cells had increased expression level of IGF2, and overexpression of IGF2 could eliminate the inhibitory effect of miR-24-3p on inflammatory damage [17]. This study found that ARDS group had increased serum IGF2 mRNA level, indicating that IGF2 may be involved in the occurrence and development of ARDS. IGF2 mRNA level increased as ARDS patients’ condition aggravated, indicating that IGF2 correlated with disease severity. A higher IGF2 level correlates with a more severe patient condition. It is speculated that the increased IGF2 level lead to an exacerbated inflammatory response, thus aggravating the inflammatory injury of the lung. Other scholars have confirmed that overexpression of IGF2 inhibits the protective effect of miR-218-5p on injury induced by oxidative modification of low-density lipoprotein (ox-LDL) in human umbilical vein endothelial cells (HUVECs), thus promoting the progression of AS, while ox-LDL stimulates HUVEC injury by promoting inflammation and inhibiting cell proliferation [18]. Further analysis of the relationship between IGF2 and prognosis revealed that patients with ARDS prognostic death had significantly higher serum IGF2 mRNA level than survivors. Moreover, the regression analysis found that IGF2 was a factor influencing prognostic death, suggesting that progression of ARDS lesions may correlate with abnormally high levels of IGF2. It is inferred that high levels of IGF2 would regulate the expression of key factors in inflammatory signaling pathway, exacerbate inflammatory response, thereby playing a role in the prognosis of ARDS. It is noteworthy that IGF2 sends signals through IGF1R, one of the key receptor tyrosine kinases involved in tumor development. IGF2 and IGF1R are considered as ideal therapeutic targets for tumors [19]. The results of this study suggested that the detection of serum FOXM1 and IGF2 mRNA levels was of great significance in predicting prognostic death of ARDS patients. Hence, in this study, the serum FOXM1 and IGF2 mRNA levels were used for prognosis evaluation. It was found that AUC prediction by combining the two was superior to FOXM1 and IGF2 mRNA alone. In addition, the combined detection could enhance the sensitivity and improve the evaluation efficiency without reducing specificity, which would help early prediction of prognostic death events in ARDS patients to guide clinical practice. In addition, Xing et al. [20] found that miR-4521 inhibited the AKT-GSK3β/Snail1 pathway by targeting IGF2 and FOXM1, thus inhibiting the epithelial mesenchymal transition process. According to the correlation analysis results in this study, FOXM1 was negatively correlated with IGF2 mRNA, suggesting that FOXM1 and IGF2 might jointly participate in the progression of ARDS through some inflammatory signaling pathway, but the specific mechanism remains unclear.

### 4.4 Conclusion

To conclude, ARDS patients had decreased serum FOXM1 mRNA level and increased IGF2 mRNA level. The two presented negative correlation, showing relevance with ARDS disease severity and 28-day prognosis. The combination of the two can be used as a reference index for evaluating the prognosis.

### 4.5 Study limitation

As for limitations of this study, the specific regulation mechanism for FOXM1 and IGF2 mRNA to participate in ARDS was not discussed, and the cases were sourced from a single center, which may lead to biased results. Sample size can be expanded to verify the conclusions. Meanwhile, the mechanism of action can be discussed from molecular pathways based on basic experiments to provide a basis for clinical diagnosis and treatment.
